# Validity of measures of pain and symptoms in HIV/AIDS infected households in resources poor settings: results from the Dominican Republic and Cambodia

**DOI:** 10.1186/1472-684X-5-3

**Published:** 2006-03-20

**Authors:** Gregory Pappas, R Cameron Wolf, Guy Morineau, Richard Harding

**Affiliations:** 1 Department of Community Health Sciences, Aga Khan University, PO Box 3500 Stadium Road Karachi, Pakistan; 2 United States Agency for International Development, Office of HIV/AIDS, Washington, DC, USA; 3 Family Health International, Cambodia; 4 Dept of Palliative Care & Policy, Guy's King's & St. Thomas' School of Medicine, London, UK

## Abstract

**Background:**

HIV/AIDS treatment programs are currently being mounted in many developing nations that include palliative care services. While measures of palliative care have been developed and validated for resource rich settings, very little work exists to support an understanding of measurement for Africa, Latin America or Asia.

**Methods:**

This study investigates the construct validity of measures of reported pain, pain control, symptoms and symptom control in areas with high HIV-infected prevalence in Dominican Republic and Cambodia Measures were adapted from the POS (Palliative Outcome Scale). Households were selected through purposive sampling from networks of people living with HIV/AIDS. Consistencies in patterns in the data were tested used Chi Square and Mantel Haenszel tests.

**Results:**

The sample persons who reported chronic illness were much more likely to report pain and symptoms compared to those not chronically ill. When controlling for the degrees of pain, pain control did not differ between the chronically ill and non-chronically ill using a Mantel Haenszel test in both countries. Similar results were found for reported symptoms and symptom control for the Dominican Republic. These findings broadly support the construct validity of an adapted version of the POS in these two less developed countries.

**Conclusion:**

The results of the study suggest that the selected measures can usefully be incorporated into population-based surveys and evaluation tools needed to monitor palliative care and used in settings with high HIV/AIDS prevalence.

## Background

Access to antiretroviral therapy (ART) has improved for people living in resource poor countries over the past year.[1] Palliative care is a critical component of these efforts because it improves the success of ART by providing symptom and pain control and because quality end-of-life care is needed by many who will die despite the increasing availability of ART. [1-5] A recent review of patient outcomes in HIV palliative care found significant improvements in the domains of pain and symptom control, anxiety, insight and spiritual wellbeing.[7]

The development of feasible and valid methods is required to assess the public health needs of communities with high HIV prevalence and to evaluate the impact of support interventions.[8,9] A range of well-validated measures have been developed and applied in resource rich settings. These measures have not been validated in resource poor countries. While no "gold standard" validation study of pain and symptoms has been devised, Palliative Outcome Scale (POS) and other measures of palliative care have been found useful in a variety of settings for program and clinical evaluation in a variety of European and United States settings. [10,11]

The objective of this paper is to study the validity of survey questionnaire items that measure aspects of palliative care, adapted from the POS, in two high HIV/AIDS prevalence populations in resource poor settings: the Dominican Republic and Cambodia. The data presented in this paper were evaluated to investigate the agreement of the relationship of measures with well developed theories related to our understanding of the relationship between chronic disease, pain, symptoms and control of pain and symptoms (construct validity). The study was also designed to evaluate the feasibility of these measures in settings with high HIV/AIDS prevalence.

## Methods

The study was conducted in two countries with mature HIV/AIDS epidemics, Cambodia and the Dominican Republic. Data were collected as part of a larger methodological study of HIV/AIDS care and support measures.[12]

### Sampling and study methods in Dominican Republic

Three peri-urban areas were studied: Santo Domingo, La Romana, and Puerto Plata. The national prevalence of HIV in Dominican Republic was 0.9 percent for person 15–49 years of age in 2002.[13]

Households were selected into the sample from a list of members of networks of people living with HIV/AIDS (PLWHA). Six established networks of PLWHA in the three peri-urban areas participated in the study. These networks were developed in poor and marginalized neighbourhoods and populations. Persons aged 18 to 59 years living in the identified network households were selected. All persons in the household who self identifies as HIV+ or were chronically ill were interviewed. Those who were chronically ill but had not disclosed their HIV status may have been HIV+ but were not known to network organizers. The questionnaire did not document HIV status. In cases of persons too sick to respond, care givers in the households acted as proxy respondents.

The survey team was trained over a one week period to administer the tool, to collect sensitive information, and to give education on HIV/AIDS. The final team included six interviewers, three supervisors, and four PLWHA guides who took interviewers to the households.

### Sampling and study methods Cambodia

Two Cambodia district were studied: rural Maung Russei district of Battambang Province and Siem Reap city. Cambodia experiences a generalized HIV/AIDS epidemic with 2.6% of person 15–59 positive.[14] The district studied were of the same approximate level of HIV prevalence compared to the national average

In Cambodia two distinct sampling strategies were used. In Maung Russei, the survey questionnaires were administered to the head of households with people living with HIV/AIDS (PLWHA). Villages to visit were selected randomly from a list of village identified through two PLWHA support groups. All households including members of the PLWHA support group in selected villages were included. In Siem Riep, respondents were recruited from outpatients at the Chronic Disease Clinic at the referral hospital. All exiting patients 18 to 59 years of age were asked to participate in the study.

No refusal to participate was recorded in either the Dominican Republic or Cambodia. This is thought to be true because the lists of potential households were constructed by network leaders who were knowledgeable about the households and listed only those households that were likely to participate. The gift of a bag of groceries also created an incentive for participation in the study. Item non-response was negligible.

### Study instrument

The study instruments included a consent form, a household roster of usual family members, age and sex, chronic illness status of persons aged 18–59. Chronic illness was defined as the inability to function or perform one's normal life role and activities due to illness and/or bed-ridden for three or more months (not necessarily consecutive) in the past year.

The questionnaire items relating to pain and other symptoms were adapted from the Palliative Outcomes Scale (POS).[15] These items asked "*In the past 30 days have you been affected by pain*": responses were *not at all*, *somewhat*, *moderate*, *severe*, *overwhelming*. For those who experienced pain respondents were further asked "*was the pain controlled*?": *no, a little*, *most of the time*, *all of the time*. The same questions were then asked regarding occurrence and control of symptoms, with examples given of nausea, coughing, diarrhea, and constipation.

Other questions inquiring into the patients' greatest need related to the illness and services received were evaluated for their feasibility for use in population based surveys. [16,17] Questionnaire items were devised to address access to external support, defined as support given from formal organizations/institutions rather than family, friends, or neighbours, services for medical care, psychological and emotional support, and other social support services.

The questionnaire and instructions were translated into Spanish and Khmer. Backwards and forwards translations were conducted. The translation teams adjusted differences by choosing appropriate wording.

### Statistical analysis

This paper assesses the construct validity of survey questionnaire items concerning pain, pain control, symptom and symptom control. Construct validity addresses the relationship between operational measurement and theoretical concepts. Our clinical experience leads us to expect that chronically ill persons will report more pain and more symptoms than not chronically persons. We also believe that control of pain should not vary by chronic disease status. That is, both the chronically ill and the not chronically ill should report the same degree of pain control at each level of reported pain. If the data are consistent with these expectations we will argue that this supports the construct validity of these questions. This should be true in settings in which pain control for the acutely or chronically ill are not available. The consistencies in the data and the agreement with observed patterns in the data that are predicted by theories are used to support construct validity.[18]

An analysis was conducted comparing measures of reported chronic illness with measures of reported pain and symptoms. A Chi square test was used to test the difference in levels of pain and symptoms between chronically ill groups and non-chronically ill groups. The selection process of subjects into the study was thought to be independent of relationships between the variables studied which is an assumption of Chi square hypothesis testing.

Mean weighted scores for pain at levels of pain control were calculated by assigning each level of pain a score. A score of one was assigned to the lowest level of pain (no pain) and a score of five to the highest level (overwhelming). A similar score system was used for symptoms.

A Mantel Haenszel test was performed to evaluate if the relationship between pain control and chronically ill status was confounded by the level of reported pain.[19] This summary Chi-square test for stratified data (chronically ill and non chronically ill) in this study was used evaluate reported pain control and symptoms control (both nominal variables) for confounding with level of reported pain and symptoms.

The analytical design of this study did not include HIV status of individuals. That is, the questionnaire did not ask about the HIV status of study participants. This study of measures of pain and symptoms sought to validate those measures in a general sense but also examine feasibility of such a study in high HIV/AIDS prevalence areas. The fact the sample persons were recruited from a network of households in which HIV positive persons were known to live according to network organizers is considered as a background characteristic of the study. The high level of HIV+ households in this study address questions about the feasibility of such a study in surveys in high prevalence settings. The data were analyzed using SAS.

## Results

A total of 354 persons were interviewed for this study. The demographic characteristics of the sample are presented in Table One. In the Dominican Republic, the sample included 87 adults not chronically ill and 82 who were found to be chronically ill. The majority of the respondents in the DR was between 25 and 39 years of age (58%) and 63% were female. The Cambodia sample had similar age and sex composition: 74% were 25 to 39 and 56% were female.

Pain and symptom prevalence and control are reported in Table Two. For the chronically ill in the Dominican Republic and in Cambodia the majority had experienced moderate to overwhelming pain (62% and 87.5% respectively) in the past thirty days. Among those who did not report chronic illness the prevalence of pain was 30% and 21% for the DR and Cambodia respectively. Among chronically ill respondents, moderate to severe symptoms were reported by 50% in the Dominican Republic and 81% in Cambodia.

Chi square tests demonstrated highly statistically significant differences in reported pain between the chronically ill group and the non-chronically ill group (Chi Squares >40, p < 0.001) in both DR and Cambodia. The same consistency and statistically significant patterns between the chronically ill and not ill respondents was observed for reported symptoms for both countries. Symptoms were more likely reported by those categorized as chronically ill compared to those who were not ill.

Table Three examines the relationship between the chronically ill and not chronically ill for pain/symptom control among those who reported pain or symptoms. Among those who reported pain the non-chronically ill were much more likely to have pain controlled. In the Dominican Republic fewer of the chronically ill (9%) had their pain completely controlled than the non chronically ill (22%) More chronically ill respondents had no pain control (9%) compared to those who were not chronically ill 6%) Similar patterns were observed in Cambodia and for symptom control.

To further evaluate the validity of the measures of pain control and symptom control we examined these variables at each level of reported pain and symptom. We expected no differences in the extent of the pain was controlled at a specific level of reported pain between the chronically ill and the non-chronically ill. The control of pain should be related to the severity of the pain and not related to whether the pain is in the context of chronic illness or acute illness. Table Four presents the mean weighted scores for pain at each level of pain for the chronically ill and the non-chronically ill. Mean weighted score for symptoms at each level of symptom control are reported in an analogous manner.

To summarize these data and test for differences we performed the Mantel- Haenszel test. In both the Dominican Republic and Cambodia, no statistical difference were observed in pain control between the chronically ill and the non-chronically ill when controlling for levels of reported pain, supporting the validity of the pain control measure (Mantel Haenszel Chi Square 2.3 p = 0.13, 2.0 p = 0.16 respectively). Similar patterns of statistical inferences were demonstrated for symptom control for the Dominican Republic, that is, the lack of statistical significant differences between the mean symptom control between the chronically ill and the not chronically ill supports the validity of this measure. In Cambodia, mean symptom scores for the chronically ill and not chronically ill were significantly different. This finding does not support the validity of the symptom control measure in Cambodia.

## Discussion

This study supports the construct validity of measures of pain, pain control, symptoms, and symptom control in two resource-poor countries. Predicted relationships between levels of pain and symptoms were observed, chronically ill respondents reporting more pain and symptoms than the non-chronically ill. Also predicted, there were no differences noted between the chronically ill and non-chronically ill for pain control measures when controlling for the level of pain. This was true in both countries, further supporting the validity of these measures.

In Cambodia the measure of symptom control did not meet the criteria for validity used in this study. Part of the sample in Cambodia included patients exiting a clinic where we expect that they had been treated for symptoms. The use of patients in a clinic may represent a limitation in the design of the study (does not meet the assumptions of the study) and does not adequately evaluate the measure of symptom control.

This study also demonstrates the feasibility of an adapted version of the POS (Palliative Outcome Scale) in settings with high prevalence of HIV/AIDS that are resource poor. No one refused to participate or to answer to specific questions. The instrument and the specific items were found to be easily administered, acceptable to interviewers and to respondents. While no formal validation study of the questionnaire items on services received or greatest need was attempted, these questionnaire items were also found to be feasible in the field. It should be noted that the success of the field work was in part due to the extensive training of the interviewers, which included information on HIV/AIDS, as well as issues revolving around stigma and discrimination. The authors of this study advocate this sort of training for all survey work that involves HIV+ respondents.

There are a number of limitations of this study that should be mentioned. Generalization of these validity findings to countries must be carefully considered. Comparisons between the two countries were not attempted. While pain and suffering are universal, most anthropological studies suggest that the experience of pain is culturally modulated.[20] Of interest, no one in the Cambodia sample reported overwhelming pain. The study translation team reported that translation of the category "overwhelming" was difficult in the Khmer language. Further research into cultural issues related to measurement of pain should be conducted to facilitate an understanding of national comparisons of palliative care.

Only construct validity is evaluated here. Face validity studies and comparisons of measures with clinically observation in resource poor countries would usefully compliment the findings of this study. The use of proxy respondents in this study for those too sick to respond may have biased the data, but is a necessary method when researching those with advanced disease or chronic debilitating illness. Bereaved relatives used as respondents for persons who have died have not been found to be good proxies to measure of pain and symptoms in the dying.[21-23]

Even though this study is not representative of a population some comment may be made on the empirical findings. There is a paucity of information on pain, pain control, services, and needs among the HIV+ in resource poor settings. The high levels of pain and the low levels of pain control are striking in these populations. The limited availability of opioids, and adequately trained staff to prescribe, pose a significant challenge in meeting the needs evidenced by this study. High levels of uncontrolled symptoms documented in this study demonstrate the need for palliative care in these populations.

## Conclusion

Despite the limitations noted here the measures evaluated in this study hold promise for use in population based household surveys and for clinical research in resource poor countries. Estimation of the pain and symptoms in HIV infected groups and in populations in general will increase our understanding of unmet needs for palliative care. Further study of the specificity and sensitivity of these measures as applied to program effectiveness and the issue of cross cultural comparisons in a number of African settings will be useful.

## Abbreviations

ART: antiretroviral therapy

DR: Dominican Republic

PLWHA: person living with HIV/AIDS

POS: palliative outcome scale

## Competing interests

The authors have no financial competing interests or non-financial competing interests that would in any way influence this study.

## Authors' contributions

GP conceived of the study, developed the statistical design, and coordination the writing of the manuscript. RCW conceived of the study, oversaw the data collection, produced the electronic data, ran preliminary data and drafted the manuscript. GM participated in the design of the study and coordinated data collection in Cambodia. RH participated in the design of the study and helped draft the manuscript. All authors read and approved the final manuscript.

## Pre-publication history

The pre-publication history for this paper can be accessed here:


